# Contaminants reach everywhere: Fish dietary samples should be surface decontaminated prior to molecular diet analysis

**DOI:** 10.1002/ece3.10187

**Published:** 2023-06-18

**Authors:** Dilli Prasad Rijal, Tanja Hanebrekke, Per Arneberg, Torild Johansen, Daniela Sint, Michael Traugott, Mette Skern‐Mauritzen, Jon‐Ivar Westgaard

**Affiliations:** ^1^ Institute of Marine Research Tromsø Norway; ^2^ Applied Animal Ecology, Department of Zoology University of Innsbruck Innsbruck Austria; ^3^ Institute of Marine Research Bergen Norway

**Keywords:** Barents Sea, bootstrapping, environmental DNA, fisheries management, predator, sampling protocol

## Abstract

Knowledge of trophic interaction is necessary to understand the dynamics of ecosystems and develop ecosystem‐based management. The key data to measure these interactions should come from large‐scale diet analyses with good taxonomic resolution. To that end, molecular methods that analyze prey DNA from guts and feces provide high‐resolution dietary taxonomic data. However, molecular diet analysis may also produce unreliable results if the samples are contaminated by external sources of DNA. Employing the freshwater European whitefish (*Coregonus lavaretus*) as a tracer for sample contamination, we studied the possible route of whitefish in beaked redfish (*Sebastes mentella*) guts sampled in the Barents Sea. We used whitefish‐specific COI primers for diagnostic analysis, and fish‐specific 12S and metazoa‐specific COI primers for metabarcoding analyses of intestine and stomach contents of fish samples that were either not cleaned, water cleaned, or bleach cleaned after being in contact with whitefish. Both the diagnostic and COI metabarcoding revealed clear positive effects of cleaning samples as whitefish were detected in significantly higher numbers of uncleaned samples compared to water or bleach‐cleaned samples. Stomachs were more susceptible to contamination than intestines and bleach cleaning reduced the frequency of whitefish contamination. Also, the metabarcoding approach detected significantly more reads of whitefish in the stomach than in intestine samples. The diagnostic analysis and COI metabarcoding detected contaminants in a higher and comparable number of gut samples than the 12S‐based approach. Our study underlines thus the importance of surface decontamination of aquatic samples to obtain reliable diet information from molecular data.

## Graphical abstract

Using an experimental approach, our study first provides evidence of how the DNA of freshwater European whitefish reaches everywhere in the gut samples of marine beaked redfish. We further demonstrate bleach‐based surface decontamination as an effective approach to reduce contamination. Finally, we urge applying a practically simple but must‐needed bleach‐based surface decontamination approach prior to molecular diet analysis in fish.

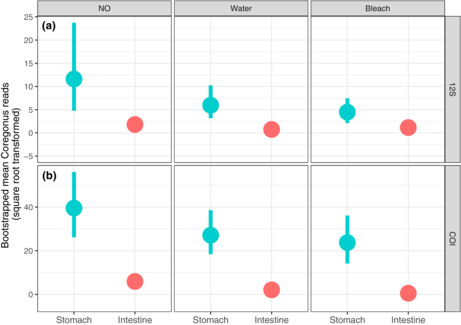

## 1 INTRODUCTION

Knowledge of trophic interactions is utterly needed for understanding the dynamics of any ecosystem and its sustainable management (Fulton et al., 2019). Being both predator and prey (Traugott et al., 2021), fish play key roles in maintaining aquatic trophic networks (Kortsch et al., 2015). Different approaches may be followed to take account of trophic interactions in the management of fish stocks (Howell et al., 2021). However, the application of such management relies on good knowledge about the trophic interactions between fish and their prey which requires large‐scale diet analyses with high taxonomic resolution.

Studies of trophic interactions have traditionally been performed by visual examination of stomach contents, which is impaired by the poor preservation of the prey and hence experts' knowledge of their identification (Traugott et al., 2021). To overcome such limitations, molecular methods based on analyses of prey DNA in stomachs, intestines, and feces have been increasingly used to study trophic interactions (King et al., 2008; Traugott et al., 2013). These methods typically detect a larger number of prey species than visual examinations (Clare, 2014) and can also be used to reveal prey diversity when prey taxonomy is unknown (Burgar et al., 2014). Once developed, the methods can be applied with low costs (Thalinger et al., 2016) and are not dependent on the observer (which may be expected for visual examinations).

Despite the enormous potential of molecular diet analysis in providing unprecedented high‐resolution taxonomic data, challenges for implementation exist. The methods are prone to contamination from other DNA sources that may produce unreliable results. Hence, contamination of gut contents by DNA of non‐food items is a source of error that must be considered in molecular diet analyses (Traugott et al., 2021). In terrestrial arthropod systems, contamination has been shown to be associated with a method based on suction sampling (King et al., 2012). With this method, individuals are squeezed together during sampling, often regurgitating. Other similar studies show that the mass collection of samples is always prone to cross‐contamination (Greenstone et al., 2012). In aquatic systems, general experience indicates that contamination may be a problem when individuals are pressed together in nets or trawls. Although such biases may be ubiquitous and unavoidable in molecular diet analysis, one should aim to reduce or manage them (Symondson & Harwood, 2014). Thus, it is crucial to minimize the contamination of samples from non‐targeted sources of DNA in all the steps involved in molecular diet analysis including sampling and other laboratory steps.

A science‐based monitoring and management system of marine ecosystems often requires information from a large number of biological samples covering large spatial and temporal scales. Trawling is one of the commonly used mass sample collecting approaches for commercial fish harvest as well as scientific sample collection. Given that all the specimens collected in the trawl are pressed together, such samples are likely to carry DNA from other species by physical contact, inhalation of water from other than their natural habitat, and predation in the net, which in turn poses challenges in identifying actual dietary elements. In such a case, it is important to apply additional cleaning approaches that potentially decontaminate the fish samples.

There are a plethora of studies dealing with biological and technical biases involved in metabarcoding as well as molecular diet analysis from wet lab to bioinformatics (see Alberdi et al., 2019; Ando et al., 2020; Ruppert et al., 2019; Thomas et al., 2014; Traugott et al., 2021; Zaiko et al., 2022 and references therein; Bohmann et al., 2022). Some studies particularly analyzed the biases involved in metabarcoding of bulk sample for diet analysis as well as impact of sample preservatives (for example Loos & Nijland, 2021; Martins et al., 2021). However, although cross‐contamination has long been recognized as one of the potential sources of bias in molecular diet analysis (King et al., 2008; Traugott et al., 2021), there has been a limited effort in understanding and mitigating biases that can arise during aquatic sample acquisition for molecular diet analysis. A few studies which attempted to mitigate such a bias were successful in terrestrial systems (Greenstone et al., 2012; Miller‐ter Kuile et al., 2021) but others either got mixed results (Oh et al., 2020) or found no effect (O'Rorke et al., 2013) in the aquatic system. Thus, we aim to study potential external biases inherent to aquatic sample collection that ultimately affect the interpretation of biodiversity assessment as well as the food composition of aquatic biota. More specifically, by considering the freshwater European whitefish (*Coregonus lavaretus*, whitefish hereafter) as a tracer for experimental sample contamination while collecting gut samples of the marine species Beaked redfish (*Sebastes mentella* Travin, redfish hereafter), we aim to:

1. Assess the performance of a diagnostic approach compared to metabarcoding in detecting cross‐contamination,
2. Study the pathways of external contamination on molecular gut content analysis,
3. Evaluate the efficacy of cleaning to reduce external contamination in gut samples, and
4. Formulate an optimized sampling protocol, applicable to molecular diet analysis of fish.

## 2 MATERIALS AND METHODS

### 2.1 Sample collection and treatment with whitefish

**FIGURE 1:**
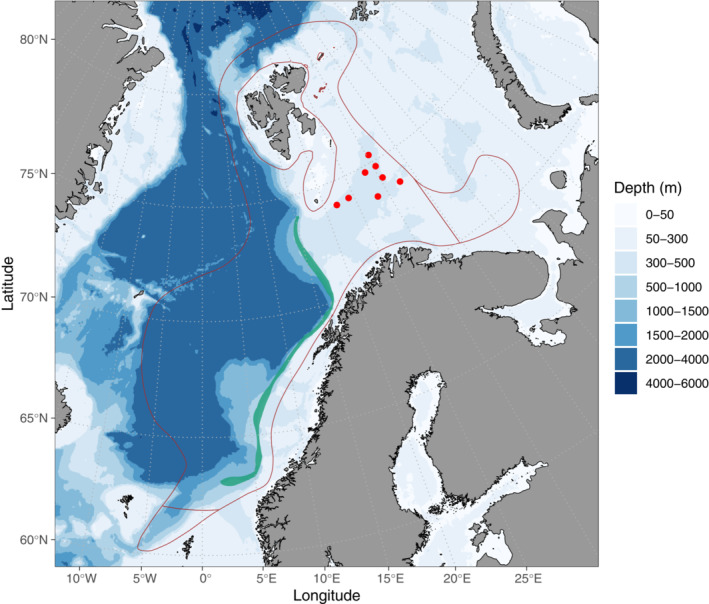
Map of the study area showing the distribution (area between brown lines) as well as breeding (green band) ranges of redfish in the Norwegian and Barents Sea along with sampling locations for molecular diet analysis (red points). Redfish distribution data source: Institute of Marine Research, Norway.

Redfish samples for molecular diet analysis were collected from eight stations from the Barents Sea (Figure 1, Data Table S1) by bottom trawling during the Barents Sea Ecosystem Survey in 2016 at the IMR R/V *Johan Hjort* using a Campelen 1800 trawl with 15 min bottom trawl time at each haul (Prozorkevich & Sunnanå, 2017). After the fish had been weighed and their length measured for other purposes at the cruise, they were made available for the current study, at which time the fish were dead. We used whitefish, a freshwater fish, as a sample contamination tracer as this species is not found in the Barents Sea. We first contaminated all the redfish samples by keeping them in a tray containing dead whitefish (contamination tray). To increase the amount of whitefish DNA released into the water, the body surface of the whitefish was incised several times with a knife. All collected redfish samples were kept in the contamination tray for 1 min, during which time the fish were moved to mimic movement in a trawl. To evaluate the effectiveness of cleaning approaches in minimizing cross‐contamination, especially physical carryover of DNA from other species and in our experiment whitefish in particular, we applied different cleaning measures on redfish abdomen before harvesting stomachs and intestines for molecular analysis. Before dissection of the redfish to collect stomach and intestine samples, the redfish surface was (i) not cleaned, (ii) cleaned with freshwater produced from desalination of seawater on the ship (hereafter referred to as water), or (iii) cleaned with water, commercial bleach, and water (bleach‐cleaning hereafter). Out of the 85 redfish samples collected, 19 samples were not cleaned, 26 samples were water‐cleaned, and 40 samples were bleach‐cleaned. A total of 85 intestines and 65 stomachs were collected from redfish samples of different cleaning categories and stored at −20°C onboard the research vessel and transferred to −80°C when the samples arrived at the laboratory. To get an overview of potential external DNA available in the treatment tray likely originating from the fish body surface, eight 50 mL falcon tubes filled with water from the contamination tray were also kept frozen with gut samples and later considered for DNA extraction as sampling control.

### 2.2 Subsampling

The stomach and intestine samples were further subsampled separately. The thawed stomach or intestine was transferred to a clean smasher bag (Seward Limited), and dissected to maximize the release of gut content using DNA‐free scissors and forceps. We added either 5 mL of 96% ethanol (7 samples) or buffer ATL (Qiagen; 78 samples) to the smasher bag (see Data Table S1), applied mild finger massage to release the gut content, removed visible tissues of redfish, and further homogenized the gut content in a smasher (SmasherTM, bioMérieux Industry) at normal speed for 60 s. We collected ca. 1.8 mL of the homogenate from each of the samples for DNA extraction. A 50 mL falcon tube half filled with milliQ water was kept open during the fish subsampling and later considered for DNA extraction as subsampling control.

### 2.3 DNA extraction

We used 180 μL of stomach or intestine homogenate, mixed with 20 μL of proteinase K, and incubated for at least 3 h or overnight for subsamples preserved in the ATL buffer. For subsamples preserved in 96% ethanol and sampling controls, we centrifuged 180 μL of stomach or intestine homogenate or water from sampling controls, removed the supernatant, added 180 μL ATL buffer and 20 μL of proteinase K, and incubated as mentioned above. We extracted DNA from all samples using Qiagen Blood & Tissue kit (Qiagen) following the manufacturer's instructions. The DNA extraction was performed in batches of 23 or 24 samples including two extraction controls per batch. We transferred 50 μL of DNA extracts into 96‐well plates and kept them frozen.

### 2.4 Polymerase chain reaction

#### 2.4.1 Diagnostic analysis

We used whitefish‐specific COI primers to amplify whitefish DNA (diagnostic analysis hereafter) in gut samples following Thalinger et al. (2016) but without Bovine Serum Albumin (BSA). As positive controls, we used whitefish DNA isolated from gill tissue diluted 100,000 times and included this in each plate (*N* = 3). All PCRs were performed in a total volume of 10 μL containing 5 μL Qiagen multiplex master mix, 0.5 μL (10 μM) primer mix, 1.3 μL dH_2_O, and 3.2 μL DNA. The thermal cycling was performed as follows: enzyme activation at 95°C for 15 min, denaturation at 94°C for 30 s, annealing at 64°C for 90 s, and extension at 72°C for 60 s with a total of 35 cycles, and a final extension at 72°C for 10 min.

PCR products were analyzed on a QIAxel Advanced instrument (Qiagen). The presence of the expected PCR products (~344 bp) with a relative fluorescence unit (RFU) value >= 0.06 was diagnosed as whitefish contamination and retained for further analysis.

#### 2.4.2 Metabarcoding

For metabarcoding of the gut content samples, we used both MiFish primers (Miya et al., 2015) targeting a hypervariable region of the 12S rRNA gene and a metazoan‐specific primer (Leray‐XT, Leray, Yang, et al., 2013; Wangensteen et al., 2018) targeting part of the mitochondrial *cytochrome c oxidase* (COI). We included 100,000 times diluted DNA mixture of *Coregonus lavaretus*, *Sebastes mentella*, *S. norvegicus*, *Gadus morhua*, *Pollachius virens*, and *Reinhardtius hippoglossoides* as a positive control in each plate as well as a positive control of each single species DNA in the second plate. We used only mixed positive controls for COI metabarcoding. We performed single‐step PCR in triplicates with fusion primers in a total of 20 μL volume that contained 10 μL Qiagen multiplex master mix, 1 μL (5 μM) primer mix, 0.16 μL (20 μg/mL) BSA, 5.84 μL dH_2_O, and 3 μL DNA. The thermal cycling was performed as follows: enzyme activation at 95°C for 10 min, denaturation at 95°C for 30 s, annealing at 60°C for 12S and 45°C for COI for 30 s, and extension at 72°C for 30 s with a total of 40 cycles for 12S and 35 cycles for COI, and a final extension at 72°C for 5 min.

### 2.5 Sequencing

All the samples were checked for PCR amplification using the QIAxel Advanced (Qiagen) instrument with the same settings as stated above. All samples were pooled per replicate plate before 100 μL from each replicate plate were pooled into a final library. To capture the targeted product size, we used 5 μL pooled amplicons and ran gel electrophoresis (2% agarose) in triplicates. The gel bands of interest were cut and collected from all three replicates and DNA was extracted and cleaned following protocol C (“DNA extraction from gel protocol”) of GeneJet Gel Extraction and DNA Cleanup Micro Kit (Thermo Fisher Scientific). We used Qubit dsDNA HS assays (Thermo Fisher Scientific) to measure the concentration of the extracted pool. The pool was diluted to a final concentration of 50 pM and spiked with 4 μL of Ion S5 Calibration Standard upon loading to the Ion Chef Instrument (Thermo Fisher Scientific). Sequencing was done on an Ion GeneStudio™ S5 System (ThermoFisher Scientific) using the Ion 530 sequencing chip and 200 bp protocol.

### 2.6 Bioinformatics

The sequencing adapters and primer sequences were trimmed from raw sequences and quality filtered by the inbuilt software of the Ion GeneStudio S5 sequencing system. The sequences were further trimmed to the expected range of the amplicon size which is typically between 163 and 185 bp (Miya et al., 2015) for 12S and ca. 313 bp for COI (Leray, Yang, et al., 2013). The length‐filtered data was further dereplicated using the *ubiuniq* function from OBITools v1.2.10 (Boyer et al., 2016). The chimeric sequences were removed using the *uchime_denovo* algorithm (Edgar et al., 2011) implemented in VSEARCH (Rognes et al., 2016). The retained sequences were clustered to generate molecular operational taxonomic units (MOTUs) using SWARM (Mahé et al., 2015) with a distance value of 3 for 12S and 13 for COI. Finally, taxonomic assignment of the MOTUs represented by 2 or more reads was performed using *ecotag* (Boyer et al., 2016) against a locally curated reference library, based on 12S and COI sequences retrieved from NCBI. All the MOTUs with the same taxonomic assignment were assumed to belong to the same taxon and therefore merged, retaining the sum of all the assigned reads. For the downstream analyses, we pulled all the metazoan sequences that had ≥90% similarity with reference sequences. Given that our interest was on *Coregonus* and *Sebastes*, the remaining taxa were grouped into potential laboratory contaminants (*Alces alces*, *Bos*, *Bos indicus*, *B. taurus*, *Gallus gallus*, *Meleagris gallopavo*, *Homo sapiens*, *Rangifer tarandus*, and *Sus scrofa*) and prey, and their respective reads and proportions were calculated (see Data Table S1). Finally, we removed the maximum number of reads detected in the PCR negative controls for respective taxa from all the samples during data analysis.

### 2.7 Statistical analysis

We found two intestinal samples (RF2283_03I and RF2303_04I) that behaved unusually among different methods and thus were considered as outliers. The overall amplification and detection of whitefish in gut samples were compared using non‐parametric one‐way ANOVA by applying the Kruskal–Wallis test followed by Dunn's test for pairwise comparison as our data did not meet the normality assumptions required for parametric tests as indicated by Shapiro–Wilk test (*W* = 0.61, *p* < .0001) of normality and an F test (*F*_₈₄,₆₄_ = 0.07, *p* < .0001) of homogeneity of variance. Thus, we used the aligned rank transform (ART) approach, which does not require normally distributed data (Wobbrock et al., 2011), with an additional multifactor contrast test using the *ARTool* package (Wobbrock et al., 2011) as an alternative to non‐parametric multifactor ANOVA (Elkin et al., 2021). We used the type “III” ANOVA test to account for the unbalanced sampling design. We also used a generalized linear model (GLM) for multiple comparisons as GLM can handle count data (O'Hara & Kotze, 2010) and has more power while analyzing data from unbalanced designs (Warton et al., 2016). Given that our response variable was the count of the number of sequences assigned to *Coregonus*, we used GLM with negative binomial distribution for pairwise comparisons. For diagnostic analysis, we converted RFU values to presence/absence data and applied binomial distribution. Diagnostic plots for GLM models (Figure A1) were generated using *DHARMa* package (Hartig, 2022) and multiple contrasts were tested using *multcomp* package (Hothorn et al., 2008). Potential impact of bleach on prey reads was also evaluated using multiple comparisons of stomach and intestines with different cleaning treatments using ART approach. We did not control significance levels for multiple comparisons, because this significantly increases the probability of dismissing real patterns (Rothman, 1990). Association between host and prey reads was visualized in log–log space for different cleaning treatments. As an additional analysis to account for the unbalanced sampling design, we calculated means and corresponding bootstrapped 95% confidence intervals for whitefish reads in stomach and intestines among different cleaning treatments using the *boot* package (Canty & Ripley, 2021; Davison & Hinkley, 1997) with 10,000 bootstrapping re‐samples. Unless stated otherwise, the *ggOceanMaps* (Vihtakari, 2022) and *ggplot2* (Wickham, 2016) packages were used for data visualization. All the analyses were performed in R version 4.1.2 (R Core Team, 2021).

## 3 RESULTS

A total of 13.74 and 28.63 million raw reads were obtained for 12S and COI metabarcoding libraries. After quality filtering, amplicon length filtering, and removal of the singletons, 5.53 (40.25%) and 14.16 (49.46%) million reads were retained for taxonomic assignment for 12S and COI metabarcoding, respectively. The final data for 12S metabarcoding that retained all the vertebrates with ≥90% similarity with reference sequences contained 5,534,815 reads (Data Table S1). For COI, we retained all the metazoans with ≥90% similarity with reference sequences and the final data contained 6,844,140 reads (Data Table S1). Mean (±SD) read for 12S and COI were 28827.16 ± 43222.22 and 35646.56 ± 41292.75, respectively. An overall 0.006% and 0.009% reads were assigned to common laboratory contaminants for COI and 12S markers respectively. A total of 6.06% and 23.16% of reads were assigned to *Coregonus*, 75.93% and 73.10% to *Sebastes*, and 18% and 3.73% to potential prey, respectively, for COI and 12S markers. Gut samples contained 0.95% and 0.12% *Coregonus*, 80.09% and 98.98% *Sebastes*, and 18.96% and 0.89% potential prey respectively for COI and 12S markers. Although gut samples were dominated by host DNA, we observed a positive association between host and potential prey reads (Figure A2). Tray water (sampling control) and gut samples shared 35% and 47% of the taxa detected by COI and 12S markers, respectively (Figure A3). Stomach and tray water samples shared 35% and 39% while intestine and tray water samples shared 22% and 43% of the taxa detected by COI and 12S markers, respectively (Figure A3). Out of the 17 taxa detected by 12S marker, nearly 59% were shared between the stomach and intestine while about 18% and 23% were unique to stomachs and intestines, respectively. Similarly, stomachs and intestines shared 54% of the taxa detected by COI marker while 43% and 3% were unique to stomachs and intestines respectively.

### 3.1 Sensitivity of diagnostic and high throughput sequencing

**FIGURE 2:**
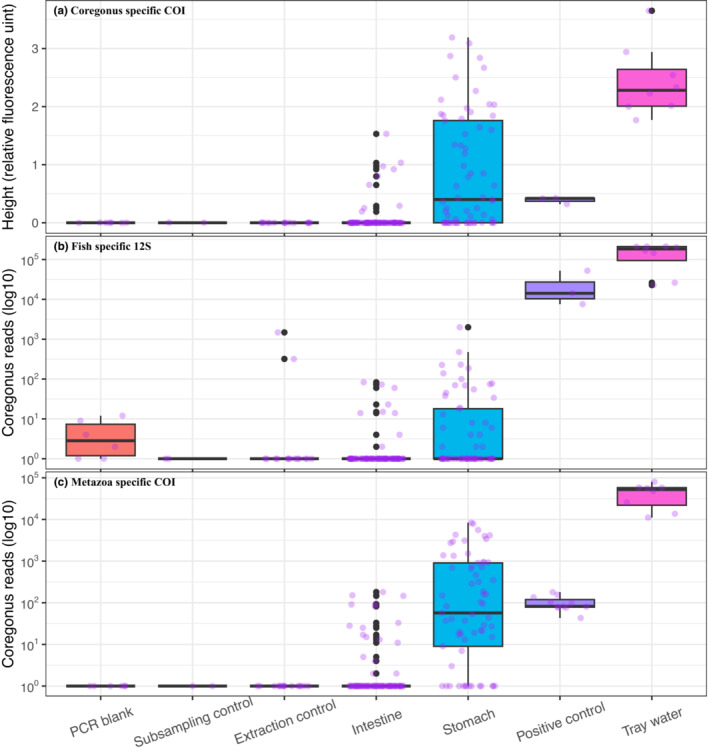
Amplification of whitefish DNA using species‐specific primer (a), and total number of reads assigned to whitefish based on metabarcoding using fish specific 12S (b) and metazoan specific COI (c) primers for different types of samples.

We found variations in the number of samples contaminated by whitefish for the three different approaches (Table A1). The diagnostic analysis mainly recorded whether whitefish DNA was present (RFU >= 0.06) or absent (RFU < 0.06) from different types of samples (Figure A4). Diagnostic analysis did not detect whitefish DNA in any of the PCR blanks (*N* = 6), sub‐sampling (*N* = 2), or extraction controls (*N* = 15; Figure 2a, Table A1). However, 12S and COI metabarcoding detected whitefish in nearly 48% and more than 4% of the control samples, respectively. Diagnostic analysis, 12S, and COI metabarcoding detected whitefish in more than 35%, 24%, and 45% of the gut samples, respectively (Table A1).

### 3.2 Fish samples get contaminated by whitefish DNA

Diagnostic analysis detected whitefish DNA in most of the stomach samples (67.7%) and a few intestine samples (10.8%, Figure 2a). The amplification strength, as indicated by RFU values, of whitefish DNA was significantly higher in stomach (mean ± SD: 0.87 ± 0.98) compared to intestine (0.08 ± 0.27; Kruskal–Wallis rank sum test: *χ* ^2^ = 51.54, *p* < .0001, Figure A5a). All positive controls (*N* = 3) and whitefish containing tray water samples (*N* = 8) showed amplification of whitefish DNA (Figure 2a). The amplification strength was highest for water samples from the collection tray (Figure 2a).

Compared to diagnostic analysis, metabarcoding based on 12S rRNA markers recorded whitefish in all sample types except subsampling controls (Figure 2b). However, metabarcoding based on metazoan‐specific COI markers showed similar results (Figure 2c) as of diagnostic analysis in terms of whitefish detection. Metabarcoding based on 12S, and COI detected white fish DNA in 41.5% and 78.5% of stomachs, respectively. Similarly, 12S and COI markers detected whitefish in 8.5% and 19.3% of the intestines respectively. An overall higher number of whitefish DNA reads were found in stomach (59.7 ± 255.1 for 12S and 923.1 ± 1805.4 for COI) than intestine (1.5 ± 7.3 for 12S and 9.5 ± 32.5 for COI) and the difference was statistically significant (Kruskal–Wallis rank sum test: *χ* ^2^ = 19.3, *p* < .0001 for 12S data and *χ* ^2^ = 59.8, *p* < .0001 for COI data, Figure A5c,e). All the tray water samples had an overall higher number of whitefish DNA reads compared to other sample types except a positive control for 12S marker (Figure 2b,c).

### 3.3 Sample cleaning reduces whitefish contamination

**FIGURE 3:**
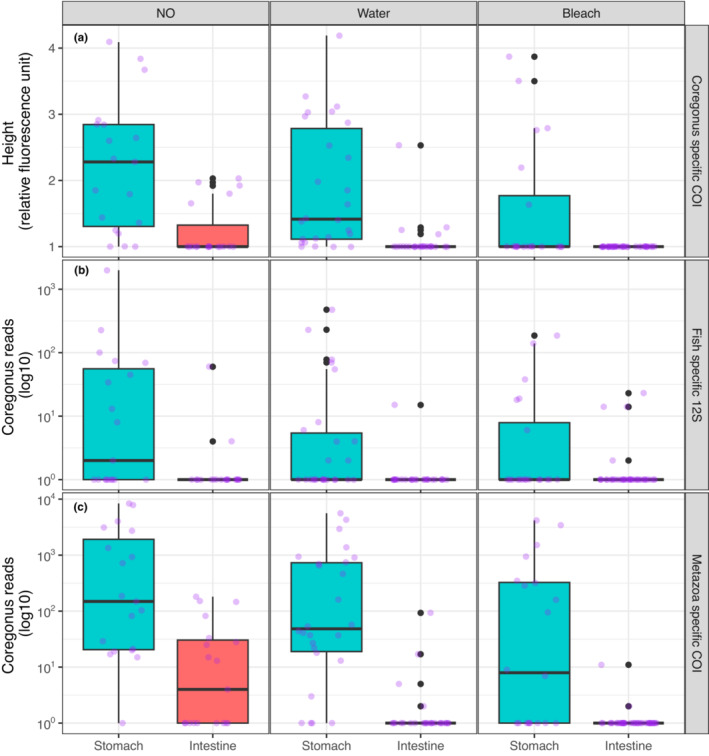
Amplification of whitefish DNA using species‐specific (a), 12S (b), and COI (c) primers from the stomach and intestine of redfish with different cleaning treatments. The whitefish DNA reads are based on metabarcoding data. See Figure A6 for zero excluded plot.

Using diagnostic PCR we found that a total of 84.2% of the stomachs from uncleaned samples were contaminated with whitefish DNA (Figure 3a, left panel) compared to only 26.3% of the intestines. Out of the 26 water‐cleaned samples, 84.6% of the stomach and 15.4% of the intestine were contaminated with whitefish DNA (Figure 3a, middle panel). The water cleaning thus diminished the strength of whitefish DNA amplification in the intestine (note reduced RFU in Figure 3a, middle panel). Out of 20 and 39 bleach‐cleaned stomachs and intestines, respectively, 30% of the stomachs and none of the intestines contained whitefish DNA (note the absence of contaminated intestine in Figure 3a, right panel).

Both GLM and non‐parametric ART approaches provided comparable results for multiple contrast tests (see Table 1), and we highlight results based on the non‐parametric test for the sake of simplicity. For the diagnostic analysis, multifactor ANOVA showed significant differences in the amplification strength of whitefish DNA between stomach and intestine (*F*_₁,₁₄₂_ = 76.1, *p* < .0001, Figure A5a), among cleaning treatments (*F*₂,₁₄₂ = 19.7, *p* < .0001, Figure A5b), and their interactions (*F*₂,₁₄₂ = 5.7, *p* < .01). Within the cleaning categories, stomachs had significantly higher whitefish DNA amplification compared to intestines (Figure 3a). Stomachs originating from samples that received all types of cleaning treatments had a significantly higher amplification than intestines from bleach‐cleaned samples (see Table 1). Whitefish DNA amplification was significantly higher in uncleaned and water‐cleaned stomachs compared to bleach‐cleaned stomachs (Table 1). Uncleaned and water‐cleaned stomachs had significantly higher amplification compared to uncleaned and water‐cleaned intestines. (Table 1, Figure 3a, Figure A5).

**TABLE 1:** Multiple comparisons of whitefish read between cleaning treatments and gut of redfish. Summary statistics are based on non‐parametric multifactor ANOVA using an aligned rank transform (ART) approach and generalized linear model (GLM).

Model	Contrast	Diagnostic				12S				COI			
		Estimate	SE	Stat	*p*‐value	Estimate	SE	Stat	*p*‐value	Estimate	SE	Stat	*p*‐value
ART	Bleach, Int ‐ Bleach, Sto	−25.15	7.50	−3.35	**.001**	−15.49	8.03	−1.93	.056	−42.29	7.71	−5.49	**.000**
ART	Bleach, Int ‐ NO, Int	−18.74	7.63	−2.46	**.015**	−0.08	8.17	−0.01	.992	−31.20	7.84	−3.98	**.000**
ART	Bleach, Int ‐ NO, Sto	−65.71	7.63	−8.61	**.000**	−33.08	8.17	−4.05	**.000**	−73.09	7.84	−9.32	**.000**
ART	Bleach, Int ‐ Water, Int	−9.62	6.91	−1.39	.166	4.61	7.41	0.62	.535	−5.84	7.10	−0.82	.412
ART	Bleach, Int ‐ Water, Sto	−60.17	6.91	−8.71	**.000**	−23.09	7.41	−3.12	**.002**	−62.70	7.10	−8.83	**.000**
ART	Bleach, Sto ‐ NO, Int	6.41	8.70	0.74	.462	15.40	9.27	1.66	.099	11.10	8.94	1.24	.216
ART	Bleach, Sto ‐ NO, Sto	−40.56	8.70	−4.66	**.000**	−17.60	9.27	−1.90	.060	−30.80	8.94	−3.45	**.001**
ART	Bleach, Sto ‐ Water, Int	15.53	8.08	1.92	.056	20.09	8.61	2.33	**.021**	36.46	8.30	4.39	**.000**
ART	Bleach, Sto ‐ Water, Sto	−35.02	8.08	−4.34	**.000**	−7.60	8.61	−0.88	.379	−20.41	8.30	−2.46	**.015**
ART	NO, Int ‐ NO, Sto	−46.97	8.81	−5.33	**.000**	−33.00	9.39	−3.51	**.001**	−41.89	9.05	−4.63	**.000**
ART	NO, Int ‐ Water, Int	9.12	8.20	1.11	.268	4.69	8.74	0.54	.592	25.36	8.42	3.01	**.003**
ART	NO, Int ‐ Water, Sto	−41.44	8.20	−5.06	**.000**	−23.00	8.74	−2.63	**.009**	−31.51	8.42	−3.74	**.000**
ART	NO, Sto ‐ Water, Int	56.10	8.20	6.84	**.000**	37.69	8.74	4.31	**.000**	67.26	8.42	7.99	**.000**
ART	NO, Sto ‐ Water, Sto	5.54	8.20	0.68	.500	10.00	8.74	1.14	.254	10.39	8.42	1.23	.219
ART	Water, Int ‐ Water, Sto	−50.56	7.53	−6.71	**.000**	−27.69	8.03	−3.45	**.001**	−56.87	7.74	−7.35	**.000**
GLM	Sto, Bleach ‐ Int, Bleach	18.72	1744.53	0.01	.991	2.71	1.14	2.39	**.017**	7.57	0.78	9.70	**.000**
GLM	Int, NO ‐ Int, Bleach	18.54	1744.53	0.01	.992	0.90	1.16	0.78	.438	4.80	0.79	6.05	**.000**
GLM	Sto, NO ‐ Int, Bleach	21.24	1744.53	0.01	.990	4.62	1.16	4.00	**.000**	8.59	0.79	10.85	**.000**
GLM	Int, Water ‐ Int, Bleach	17.86	1744.53	0.01	.992	−0.90	1.08	−0.83	.406	2.71	0.73	3.69	**.000**
GLM	Sto, Water ‐ Int, Bleach	21.27	1744.53	0.01	.990	3.29	1.05	3.13	**.002**	7.84	0.73	10.76	**.000**
GLM	Int, NO ‐ Sto, Bleach	−0.18	0.71	−0.26	.798	−1.81	1.31	−1.39	.166	−2.77	0.84	−3.32	**.001**
GLM	Sto, NO ‐ Sto, Bleach	2.52	0.80	3.17	**.002**	1.91	1.30	1.46	.144	1.02	0.83	1.22	.221
GLM	Int, Water ‐ Sto, Bleach	−0.86	0.73	−1.17	.240	−3.61	1.24	−2.92	**.004**	−4.86	0.78	−6.23	**.000**
GLM	Sto, Water ‐ Sto, Bleach	2.55	0.73	3.49	**.000**	0.57	1.21	0.48	.635	0.27	0.78	0.34	.731
GLM	Sto, NO ‐ Int, NO	2.70	0.82	3.31	**.001**	3.72	1.32	2.81	**.005**	3.79	0.85	4.48	**.000**
GLM	Int, Water ‐ Int, NO	−0.68	0.75	−0.90	.370	−1.80	1.26	−1.43	.153	−2.09	0.79	−2.64	**.008**
GLM	Sto, Water ‐ Int, NO	2.73	0.75	3.63	**.000**	2.39	1.23	1.94	.053	3.04	0.79	3.86	**.000**
GLM	Int, Water ‐ Sto, NO	−3.38	0.83	−4.06	**.000**	−5.52	1.26	−4.40	**.000**	−5.88	0.79	−7.43	**.000**
GLM	Sto, Water ‐ Sto, NO	0.03	0.83	0.04	.970	−1.33	1.23	−1.08	.278	−0.75	0.79	−0.96	.338
GLM	Sto, Water ‐ Int, Water	3.41	0.77	4.44	**.000**	4.19	1.16	3.62	**.000**	5.13	0.73	7.04	**.000**

*Note*: Negative binomial distribution was used in GLM for COI and 12S markers, and binomial distribution for diagnostic analysis. *T*‐test and *z*‐test were used for ART and GLM models, respectively. Statistically significant *p*‐values are indicated in boldface. Sto, Stomach and Int, Intestine.

Metabarcoding based on 12S rRNA markers detected whitefish DNA in both stomachs and intestines that had different cleaning treatments. A total of 52.6%, 42.3%, and 30% of the uncleaned, water‐cleaned, and bleach‐cleaned stomachs were contaminated with whitefish DNA. Similarly, whitefish contamination was found in 10.5%, 3.9%, and 10.8% of the uncleaned, water‐cleaned, and bleach‐cleaned intestines. An overall higher number of whitefish DNA reads were detected in the stomachs than intestines within all cleaning treatments (Figure 3b). Regarding the contamination in stomachs, the highest number of whitefish DNA reads (134.5 ± 453.7) was found in uncleaned, a moderate number of reads (35.5 ± 101.8) in water cleaned, and the lowest number of reads (20.00 ± 49.9) in bleach cleaned samples. The mean whitefish DNA read was highest (3.3 ± 13.5), moderate (1.3 ± 4.6), and lowest (0.5 ± 2.8) in the intestines of uncleaned, bleach‐cleaned, and water‐cleaned samples respectively.

For 12S metabarcoding, multifactor ANOVA showed significant differences in the number of reads of whitefish DNA between intestine and stomach (*F*₁,₁₄₁ = 85.4, *p* < .0001), cleaning treatments (*F*₂,₁₄₁ = 4.0, *p* < .05), and interactions of gut types and cleaning treatments (*F*₂,₁₄₁ = 4.7, *p* < .05). We found a significantly higher number of whitefish DNA reads in the uncleaned stomachs compared to the uncleaned, water‐cleaned, and bleach‐cleaned intestines. Similarly, water‐cleaned stomachs had significantly higher numbers of whitefish DNA reads compared to both water‐ and bleach‐cleaned intestines (Table 1, Figure 3b). We also found that the overall number of whitefish reads was not significantly different among cleaning treatments (Figure A5).

Metabarcoding based on COI markers detected whitefish DNA in 94.7%, 84.6%, and 55% of the uncleaned, water‐cleaned, and bleach‐cleaned stomachs, respectively, and in 52.6%, 15.4%, and 5.3% of the uncleaned, water‐cleaned, and bleach‐cleaned intestines, respectively. An overall higher and similar number of whitefish DNA reads was detected in the stomachs than intestines within all cleaning treatments (Figure 3c). Regarding the contamination in stomachs, the mean whitefish DNA reads were highest (1561.1 ± 2598), moderate (734.4 ± 1408.9), and lowest (562.4 ± 1173.6) for uncleaned, water‐, and bleach‐cleaned samples. The mean whitefish DNA read was highest (35.2 ± 58.5) in uncleaned intestines followed by water‐cleaned (4.4 ± 18.2), and bleach‐cleaned (0.3 ± 1.6) intestines.

For COI metabarcoding, multifactor ANOVA showed significant differences in the number of reads of whitefish DNA between intestine and stomach (*F*₁,₁₄₂ = 118.4, *p* < .0001), cleaning treatments (*F*₂,₁₄₂ = 12.1, *p* < .0001), and interactions of gut types and cleaning treatments (*F*₂,₁₄₂ = 7.7, *p* < .001). As found in the 12S, the number of whitefish DNA reads was significantly higher in the uncleaned stomachs compared to the uncleaned, water‐cleaned, and bleach‐cleaned intestines, and water‐cleaned stomachs had significantly higher numbers of whitefish DNA reads than both water‐ and bleach‐cleaned intestines (Figure 3c, Table 1, Figure A6). Out of the 15 contrast comparisons, 12 comparisons showed statistically significant differences in the whitefish DNA reads. However, we observed similar numbers of whitefish DNA reads in bleach‐cleaned stomachs and uncleaned intestines, uncleaned and water‐cleaned stomachs, and bleach‐cleaned and uncleaned intestines. This similarity between gut types and cleaning treatments was supported by all three markers.

The potential prey reads did not differ significantly for all the comparisons between gut and cleaning treatments for 12S metabarcoding (Table A2). In the case of COI metabarcoding, the number of prey reads was significantly higher in bleach‐cleaned intestines compared to both uncleaned‐ and water‐cleaned intestines (Table A2).

**FIGURE 4:**
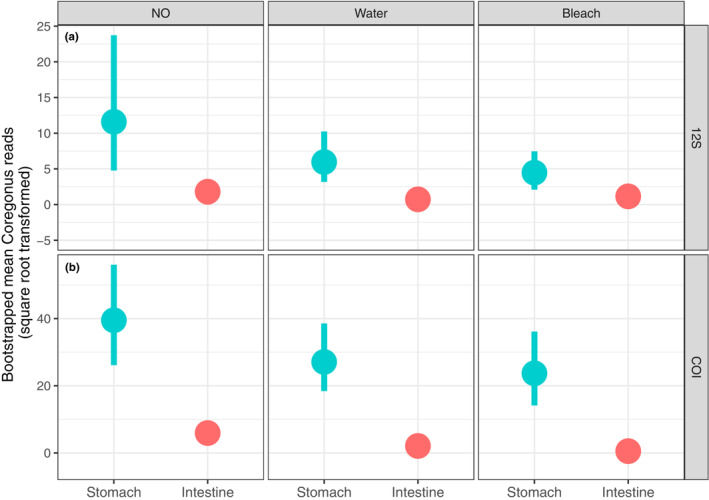
Mean number of whitefish reads detected by metabarcoding of redfish gut samples with different cleaning treatments using (a) 12S and (b) COI markers. The error bars indicate a 95% bootstrapped confidence interval of mean. Note too little variation in the case of intestines. See Figure A7 for untransformed *y*‐scales.

The bootstrapped mean whitefish DNA reads were always higher in the stomachs compared to the intestine (Figure 4, Figure A7) and generally decreased along the cleaning gradient, particularly for stomachs. The difference was distinct for uncleaned and water‐cleaned samples, and rather subtle for bleach‐cleaned samples in the case of 12S metabarcoding (Figure 4a). In the case of COI metabarcoding, the bootstrapped mean whitefish DNA reads were distinctly higher for the stomach compared to the intestine within all cleaning categories (Figure 4b).

## 4 DISCUSSION

### 4.1 Sensitivity of diagnostic and high throughput sequencing

Sample cross‐contamination by DNA may cause serious biases in the interpretation of food components based on molecular diet analysis (Traugott et al., 2021). One of the aims of this study was to compare the whitefish‐specific diagnostic analysis to high throughput metabarcoding in terms of sensitivity in contamination detection. The COI metabarcoding detected whitefish in the highest number of gut samples followed by diagnostic analysis. The close similarity of diagnostic and COI‐based results might be due to the same target gene and comparable amplicon length (344 and 315 bp, Leray, Yang, et al., 2013; Thalinger et al., 2016). Compared to diagnostic and COI metabarcoding, 12S metabarcoding detected whitefish in the least number of gut samples.

The discrepancies in the detection among methods might be due to the differences in the sensitivity of the approaches and amplification biases associated with different primers used in each method (see Browett et al., 2021; Hansen et al., 1998) as well as stochastic amplification (Kebschull & Zador, 2015). The lowest detection in the case of 12S marker may be due to the PCR biases originating from primer‐template mismatches as MiFish primers have been reported to under‐represent several freshwater fishes (Miya et al., 2020). It is also a fact that the predator DNA is generally present in both good quality and quantity in the gut samples (Drake et al., 2022; Leray, Yang, et al., 2013), and very low amounts of template DNA of whitefish might have been outcompeted during the PCR by the dominant predator DNA (sensu Cuff et al., 2022; Kebschull & Zador, 2015; Paula et al., 2015). Although there was a positive association between host and prey DNA reads, and we retained a usable amount of prey reads for further analyses, the proportion of reads could be increased using host‐specific blocking primers (Homma et al., 2022; Leray, Agudelo, et al., 2013).

The diagnostic analysis targeted COI markers with whitefish‐specific primers and we can expect the highest number of detections by this approach. Note that we set the threshold of >=0.06 RFU as suggested by Thalinger et al. (2016) to consider the presence of whitefish in a sample. The potential reason for detecting whitefish in fewer samples than for the general COI metabarcoding primer may be due to the removal of samples with a lower signal which might have been otherwise detected by the highly sensitive metabarcoding. In the case of general metabarcoding primers that targeted the COI gene of metazoa, there might have been equal opportunity of amplification for all the templates as the primer has no known preferential amplification over whitefish and redfish.

Whitefish detection in higher numbers of controls by the 12S approach may be due to several factors including DNA extract contamination, cross‐contamination during PCR or amplicon handling. The detection of the predator in all types of controls by both approaches indicates that contamination is highly likely and unavoidable, as also noted by others (Sepulveda et al., 2020), particularly when the subsampling and extraction area is dominated by the predator DNA. However, we can rule out the hypothesis that our DNA extracts got contaminated during handling, as we ran all the analyses from the same DNA extracts step by step starting with the diagnostic method (no whitefish in controls), then 12S metabarcoding (whitefish detected in all type of controls), and finally the COI metabarcoding (whitefish in one control represented by single read). Thus, the whitefish detected in most of the controls in the case of 12S metabarcoding entered the samples during library preparation, either due to cross‐contamination during PCR or minor contamination through aerosol or carry‐over from the pipette while handling the amplicons. Given that contaminants in the controls were not detected by two of the three methods used, our results and their interpretations should be reliable and reproducible.

As we have used contaminant‐specific primers in the diagnostic analysis and the results show a good match with the COI metabarcoding, we emphasize that results based on these two approaches are reliable and should be preferred over the 12S‐based method. It is more reasonable to use species‐specific diagnostic analysis if the aim is to detect a specific contaminant as diagnostic analyses are robust and reproducible (Rennstam Rubbmark et al., 2019; Traugott et al., 2021). If a tracer has been used to track the route of contamination, frequency and read statistics of the tracer should be used further to inform bioinformatic and statistical analyses to mitigate additional biases due to contamination.

### 4.2 Biological samples are likely to get contaminated in the trawl

Our findings indicate that fish samples collected by trawl are highly susceptible to cross‐contamination from other sources of environmental DNA. In our case, more than 45% of the gut samples were contaminated with whitefish DNA by being exposed to an environment containing whitefish DNA. This indicates that cross‐contamination is a pervasive issue in molecular diet analysis as also revealed by several empirical studies from different systems (De la Cadena et al., 2017; Galan et al., 2018; Greenstone et al., 2012). Such contamination seems to be potentially manageable in terrestrial systems (Greenstone et al., 2012; Remén et al., 2010; Sow et al., 2020). However, cross‐contamination is unavoidable during aquatic sample collection, particularly while using mass collection equipment such as trawl. Biological materials are alive in the trawl, are pressed against each other, and likely engulf materials regurgitated by other organisms, and inhale water from other areas than their natural habitats, making it easier for DNA from other organisms to enter the body of the predator. In such a situation, although there is no practical way to avoid contamination, it is important to reduce and manage the biases/noises as much as possible (Sepulveda et al., 2020; Traugott et al., 2021).

We detected whitefish in nearly half of the gut samples despite the short exposure (ca. 1 min) of fish samples to a whitefish‐containing tray. There may be 100 s of taxa collected and pressed together in a trawl for a relatively longer period making biological samples highly vulnerable to cross‐contamination from non‐targeted DNA. If this source of bias is not reduced and managed, the fresh and intact DNA of non‐food taxa, not affected by enzymatic reactions inside the host's gut, may get preferentially amplified, dominating the actual prey taxa in the amplicon pool. If we base our decision on the dominant taxa, using the number of reads, then our inferences will be seriously biased and unreliable. Thus, it is crucial to implement a surface decontaminating approach that can circumvent or at least dramatically reduce non‐target DNA reaching to gut samples.

### 4.3 Sample cleaning reduces contamination from non‐target sources

The highest proportion of uncleaned stomachs were contaminated with whitefish DNA indicating that cross‐contamination may likely occur by physical contact and carryover of DNA as stomachs are physically closer to exposure to contaminants than intestines. If diet composition is merely inferred based on the detection, we would have concluded whitefish as one of the most frequently eaten prey. Such a conclusion would mislead the actual prey identification, severely affecting management decisions (Traugott et al., 2021). However, physical carryover of contaminants can easily be reduced, if not completely removed, by simply rinsing with water (but see O'Rorke et al., 2013) and more effectively by bleach cleaning the surfaces of biological samples (Greenstone et al., 2011; Remén et al., 2010). On the contrary, bleach‐based cleaning has been suggested to be used cautiously as soluble bleach is more permeable to aquatic than terrestrial animals (see O'Rorke et al., 2013) and it may severely degrade the DNA quality of samples. Contrary to expectations, we found higher prey reads in bleach‐cleaned intestines than in uncleaned and water‐cleaned intestines and there was no indication of prey DNA degradation in bleach‐cleaned stomachs. It is likely that the thick tissue of redfish is less permeable to bleach so that the quality of prey DNA remained intact in this case. However, such a risk may be highly likely in the case of other delicate aquatic organisms such as spiny lobster larvae and alike (O'Rorke et al., 2013). Thus, it is important to consider the delicacy of target aquatic organisms to the bleach permeability prior to applying bleach‐based cleaning treatment.

We found clear differences in the level of contaminant among samples that had been exposed to different cleaning treatments, with contamination generally decreasing along the cleaning gradient from no cleaning to water cleaning and finally bleach cleaning of the fish surface prior to collection of the stomach and intestines. It is impractical to hand‐pick the biological samples of interest, especially from marine environments, as suggested by others (King et al., 2008) to minimize the cross‐contamination. However, we consider surface cleaning of fish samples by bleach as a simple and practically feasible approach to reduce the overall amount of contaminant as also reported in other systems (Greenstone et al., 2012; Miller‐ter Kuile et al., 2021; Oh et al., 2020) and while assessing the impact of intra‐specific DNA contamination in population genetic analysis (Petrou et al., 2019). Once the samples are cleaned, the number of reads assigned to contaminants gets lowered and the minimum read threshold set during the bioinformatic pipeline may already remove the potential contaminants.

Both the diagnostic and COI metabarcoding approaches indicate significant removal of the contaminant by bleach cleaning. Although the number of samples where whitefish was detected by 12S metabarcoding varied compared to other methods, it also indicated a positive effect of sample cleaning on contamination removal. Our results unanimously show that fish body surfaces should be cleaned to get less biased results from molecular diet analysis. Thus, in line with others (e.g. Greenstone et al., 2012; Miller‐ter Kuile et al., 2021), we recommend surface decontamination of fish samples by water and bleach prior to gut sample collection to minimize the cross‐contamination from non‐target sources of DNA.

### 4.4 Fish sample acquisition for molecular diet analysis

Our study clearly shows that contaminants can dominate the stomach and reach the intestine even within a very short time of exposure. Note that we have explored the route of a known contaminant. However, there might be several taxa of contaminants on the body surface of fish that we do not know. It is important to emphasize that the contaminants detected in this study are assumed to have originated from the surface of the fish samples. To our knowledge, there is a lack of information about the water movement in dead fishes. Thus, we could not rule out the possibility of contamination of samples by direct water movement into the gut of fishes. If water movement is possible in (almost) dead fish, then we expect the stomach to contain more contaminants than the intestine as external DNA passes through the stomach before reaching the intestine. We detected a consistently higher number of *Coregonus* reads in the stomach than intestine supporting the water movement hypothesis in supposedly dead fish. Further, a high overlap of taxa detected between gut and tray water samples may also indicate that water from the collection tray might have entered the gut. This type of contamination directly through the digestive tract cannot be removed by external cleaning and any effort to clean internal contaminants from the gut will also adversely affect the prey DNA. As an alternative one can sample intestines to reduce contaminants as they seem to be less susceptible to external contamination. However, it is also important to consider unique taxa present in stomachs which seem to be quite high as reflected by the COI marker. Thus, although there is no ideal way to control and reduce contaminants from the digestive tract, mixing both stomach and intestines may dilute the overall amount of external DNA and also maximize prey detection if there are any unique taxa in stomachs and intestines. When it comes to surface decontamination, detection of significantly lower number of *Coregonus* reads in bleach‐cleaned guts compared to uncleaned or water‐cleaned guts indicates that cleaning treatment is effective in reducing contaminants from one of the sources, namely body surface. Thus, it is crucial to apply cleaning treatment to decrease the overall number of contaminants in the gut prior to molecular diet analysis.

Let us imagine that certain contaminants enter the body of fish through water movement as well as from the surface while handling the samples. As a combined effect of these two processes gut content of the target fish may be dominated by the contaminants outcompeting actual prey taxa during PCR. Thus, by employing a surface decontamination approach, the level of contamination can be minimized, and contaminants get penalized during both PCR and bioinformatics steps, ultimately removing the rare contaminants. Thus, in addition to recommended best practices for DNA‐based approaches (King et al., 2008; Traugott et al., 2021), we suggest the following (see Protocol A1 in the Appendix for the detailed protocol) while acquiring fish samples:

1. Establish subsampling controls in the dissection room and also take swab samples of individual fish surfaces.
2. Rinse each sample with target DNA‐free water, and 1% sodium hypochlorite (leave it for 5–10 min to make it effective), and finally rinse thoroughly with sterile water.
3. Freeze the samples if dissection is not possible in the field or dissect.
4. If dissected, collect both the stomach and intestine contents in a target‐DNA‐free smasher bag, and add an appropriate volume of 70%–90% ethanol or ATL buffer to make homogenate and homogenize samples by mechanical smasher or by manually massaging the bag.
5. Take subsamples from the homogenate in an appropriate volume and numbers for DNA extraction, and freeze.

## 5 CONCLUSIONS

Our results clearly indicate that the biological samples collected for molecular diet analysis using mass‐collecting tools such as trawl are prone to contamination. We show that contaminants reach everywhere in the gut samples; however, their amplification strength and frequency of contaminated samples are significantly reduced by surface decontamination. We also provide brief guidelines for fish sample acquisition for molecular diet analysis that minimizes the biases from external contamination and maximizes prey capture. We are aware that diet detection gets affected by other factors such as mass of prey consumed and duration of prey consumption (Schattanek et al., 2021). Detection of contaminants in very high frequency of gut samples may be related to the freshly released DNA from whitefish not affected by the enzymatic reactions compared to potentially degraded DNA of prey which has been reported to be impossible to detect after a few days of experimental feeding (Holman et al., 2021; Jo et al., 2017; Thuo et al., 2019). We cannot rule out the fact that the effect of contaminants may be less severe in the natural settings compared to our experimental approach where we incised whitefish to release DNA. Although we have not explored how much DNA from contaminants entered the stomach and intestine directly, none of the cleaning strategies will be effective to remove contaminants from the predators' digestive tract completely. We also emphasize that this study is not meant to provide a full spectrum of diets of redfish; rather focuses on a sampling approach to minimize the likely biases and maximize prey catch in a molecular diet analysis framework. The bleach‐based decontamination approach has been demonstrated to work effectively in terrestrial arthropod systems (Briem et al., 2018; Greenstone et al., 2012). However, permeability of bleach to specific organisms should be assessed prior to the application of bleach‐based treatments as bleach may severely degrade the prey DNA (see O'Rorke et al., 2013). Our work is probably the first experiment attempting to remove contaminating DNA in fish‐based systems. Thus, we emphasize that further work is needed to improve and establish a decontamination process relevant to aquatic/fish‐based systems. We hope this study helps to improve the DNA‐based analysis of the diet of fish and stimulates further research on how to treat fish dietary samples to minimize the effect of contaminating DNA.

## AUTHOR CONTRIBUTIONS

**Dilli Prasad Rijal:** Data curation (lead); formal analysis (lead); investigation (lead); methodology (equal); validation (lead); visualization (lead); writing – original draft (lead); writing – review and editing (lead). **Tanja Hanebrekke:** Investigation (equal); methodology (equal); validation (supporting); visualization (supporting); writing – original draft (supporting); writing – review and editing (supporting). **Per Arneberg:** Conceptualization (lead); data curation (equal); funding acquisition (equal); investigation (supporting); methodology (lead); writing – original draft (supporting); writing – review and editing (supporting). **Torild Johansen:** Conceptualization (equal); funding acquisition (supporting); writing – original draft (supporting); writing – review and editing (supporting). **Daniela Sint:** Conceptualization (supporting); investigation (supporting); writing – original draft (supporting); writing – review and editing (supporting). **Michael Traugott:** Conceptualization (supporting); writing – original draft (supporting); writing – review and editing (supporting). **Mette Skern‐Mauritzen:** Funding acquisition (lead); project administration (lead); writing – original draft (supporting); writing – review and editing (supporting). **Jon‐Ivar Westgaard:** Conceptualization (equal); funding acquisition (equal); investigation (supporting); methodology (equal); supervision (lead); writing – original draft (supporting); writing – review and editing (supporting).

## CONFLICT OF INTEREST STATEMENT

None declared.

## Supporting information


Appendix A1.

Table S1.

## Data Availability

The raw sequencing reads have been deposited in the GenBank Sequence Read Archive at project accessions PRJNA974684, and sample accessions SAMN35560076 to SAMN35560261 for 12S marker and SAMN35560364 to SAMN35560555 for COI marker along with associated metadata. The data required to replicate the analyses of this paper are included as the Supporting Information.
