# Supplementary material for: Contaminants reach everywhere: Fish dietary samples should be surface decontaminated prior to molecular diet analysis
Source: Ecol Evol. 2023 Jun 18;13(6):e10187. doi: 10.1002/ece3.10187 (PMC10277604; doi:10.1002/ece3.10187)
Supplement: Supplementary file 1 — Appendix A1. [file ECE3-13-e10187-s002.docx]

# Appendix

**Table A1** Number of whitefish contaminated samples under different sample categories and methods. Only 39 bleach-cleaned intestines are considered for 12S metabarcoding. The last column indicates the number of samples where *Sebastes* was detected using 12S and COI metabarcoding.

| **Sample types** | **Cleaning** | **Total Samples** | **Method** | | | **Sebastes**  **12S/COI** |
| --- | --- | --- | --- | --- | --- | --- |
|  |  |  | **Diagnostic** | **12S Metabarcoding** | **COI Metabarcoding** |  |
| PCR control |  | 6 | 0 | 4 | 0 | 3/1 |
| Extraction control |  | 15 | 0 | 7 | 1 | 12/8 |
| Subsampling control |  | 2 | 0 | 0 | 0 | 2/1 |
| Stomach | No | 19 | 16 | 10 | 18 | 18/19 |
|  | Water | 26 | 22 | 11 | 22 | 25/26 |
|  | Bleach | 20 | 6 | 6 | 11 | 20/20 |
| Intestine | No | 19 | 5 | 2 | 10 | 18/16 |
|  | Water | 26 | 4 | 1 | 4 | 25/25 |
|  | Bleach | 40 | 0 | 6 | 3 | 38/33 |

Table A2 Multiple comparisons of potential prey reads between cleaning treatments and gut types of redfish. Summary statistics are based on non-parametric multifactor ANOVA using aligned rank transform (ART) approach. Statistically significant p-values are indicated in boldface.

| **Contrasts** | **Markers** | **Estimate** | **SE** | **t-ratio** | **p-value** |
| --- | --- | --- | --- | --- | --- |
| Bleach,Intestine - NO,Intestine | 12S | -5.89 | 11.66 | -0.51 | 0.614 |
| Bleach,Intestine - Water,Intestine | 12S | -15.38 | 10.54 | -1.46 | 0.147 |
| Bleach,Stomach - NO,Stomach | 12S | -23.93 | 13.41 | -1.78 | 0.076 |
| Bleach,Stomach - Water,Stomach | 12S | -6.69 | 12.45 | -0.54 | 0.592 |
| NO,Intestine - Water,Intestine | 12S | -9.49 | 12.63 | -0.75 | 0.454 |
| NO,Stomach - Water,Stomach | 12S | 17.24 | 12.63 | 1.36 | 0.174 |
| Bleach,Intestine - NO,Intestine | COI | 33.19 | 9.95 | 3.34 | **0.001** |
| Bleach,Intestine - Water,Intestine | COI | 25.82 | 9.00 | 2.87 | **0.005** |
| Bleach,Stomach - NO,Stomach | COI | 16.45 | 11.44 | 1.44 | 0.153 |
| Bleach,Stomach - Water,Stomach | COI | 6.17 | 10.62 | 0.58 | 0.562 |
| NO,Intestine - Water,Intestine | COI | -7.37 | 10.78 | -0.68 | 0.495 |
| NO,Stomach - Water,Stomach | COI | -10.28 | 10.78 | -0.95 | 0.342 |


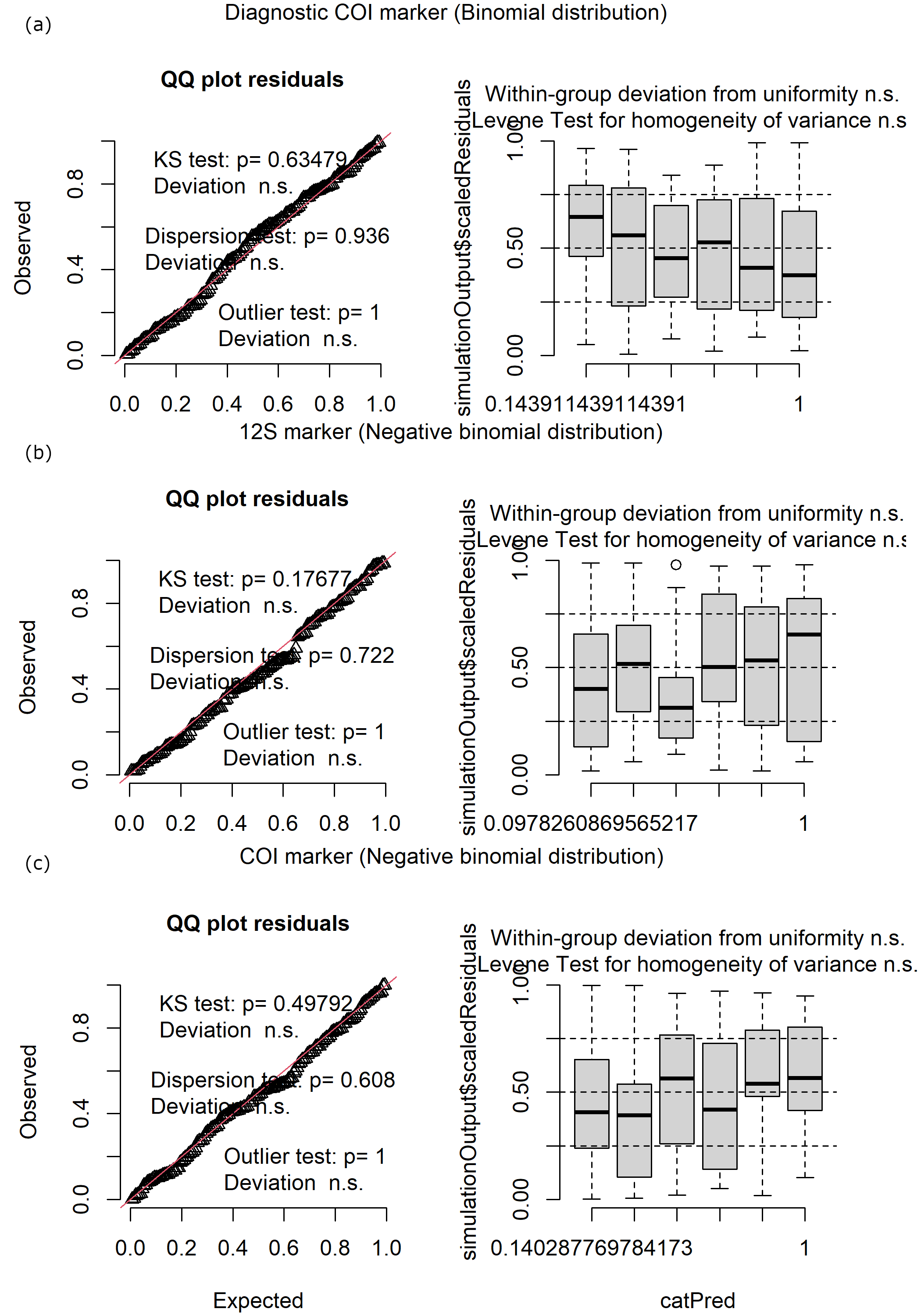


Figure A1 Residuals and other model verification statistics of generalised linear models for (a) diagnostic COI, (b) 12S, and (c) COI markers.


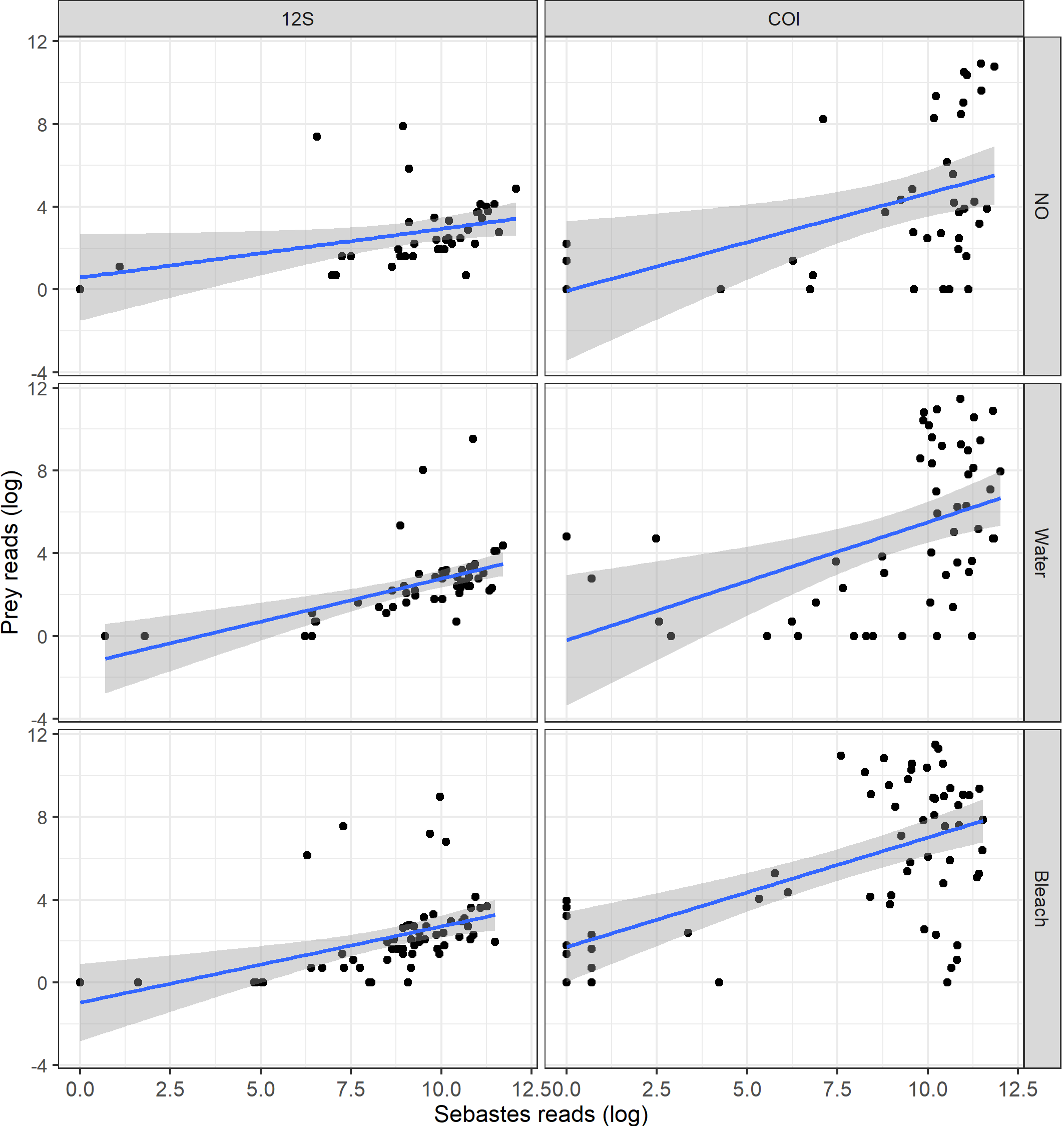


Figure A2 Positive association between *Sebastes* and prey reads.


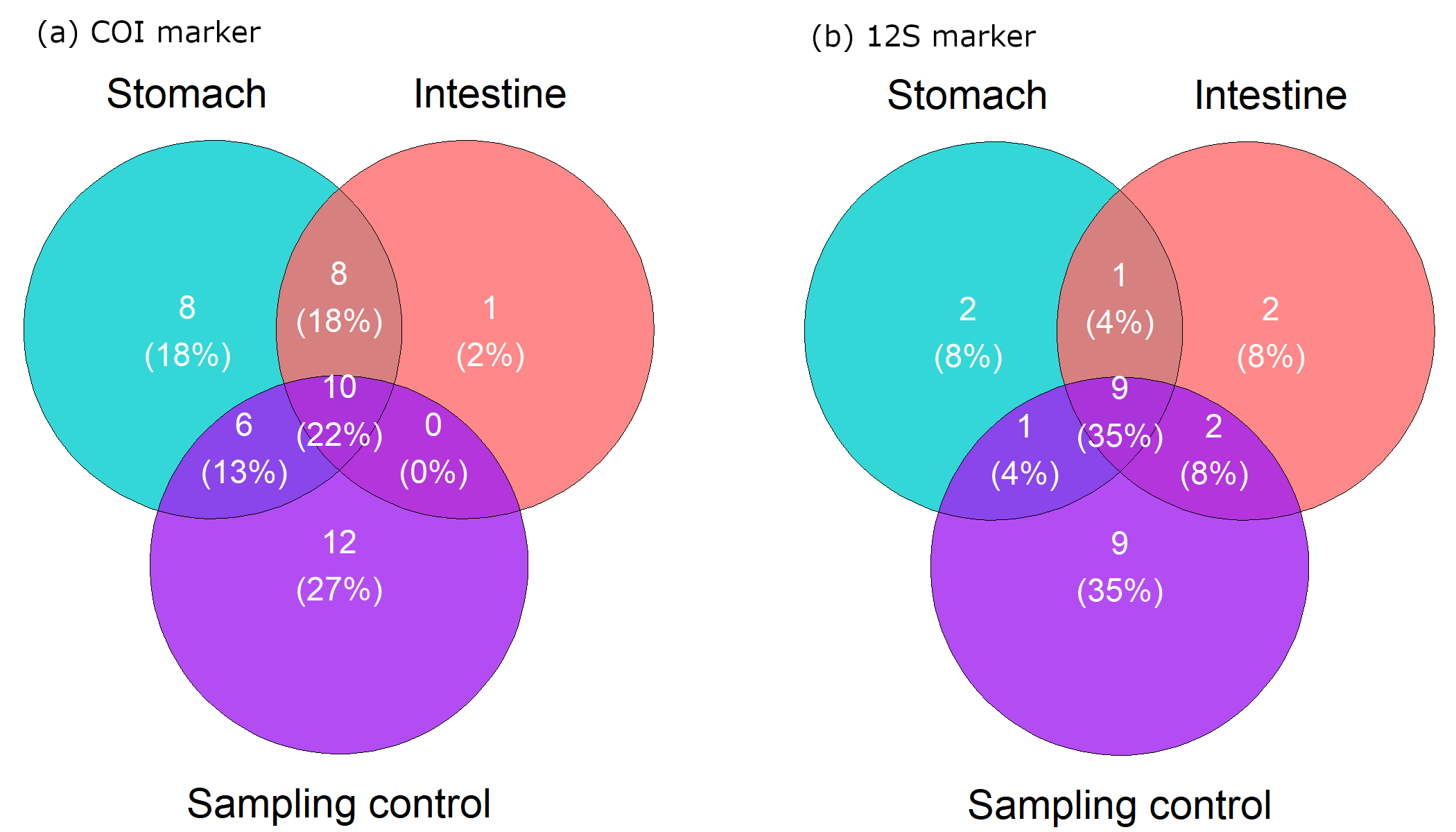


Figure A3 Number of shared and unique taxa in stomach, intestine, and tray water samples (sampling control) detected by (a) COI, and (b) 12S markers.

**
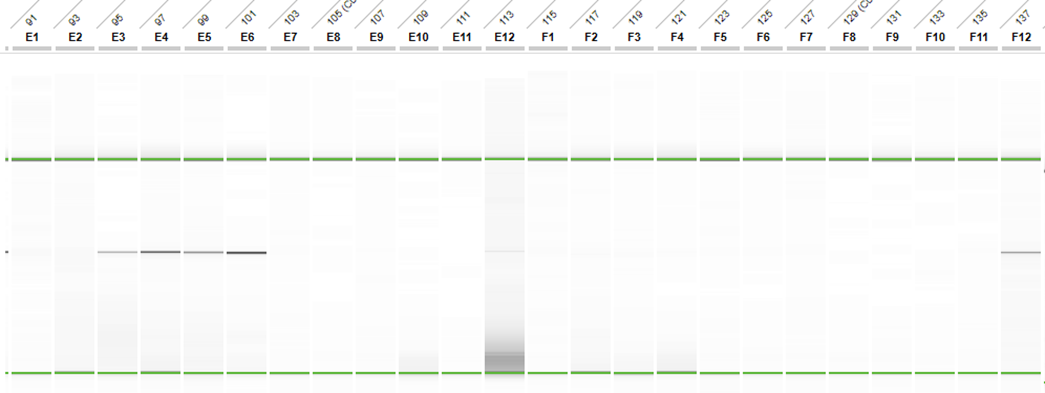

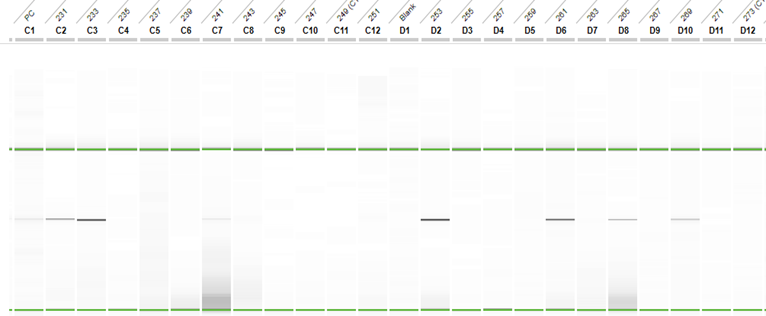
**

**Figure A4** Screenshots of diagnostic analysis illustrating the detection of the whitefish (black bands) along with scale bars (green bands) for some of the gut samples.


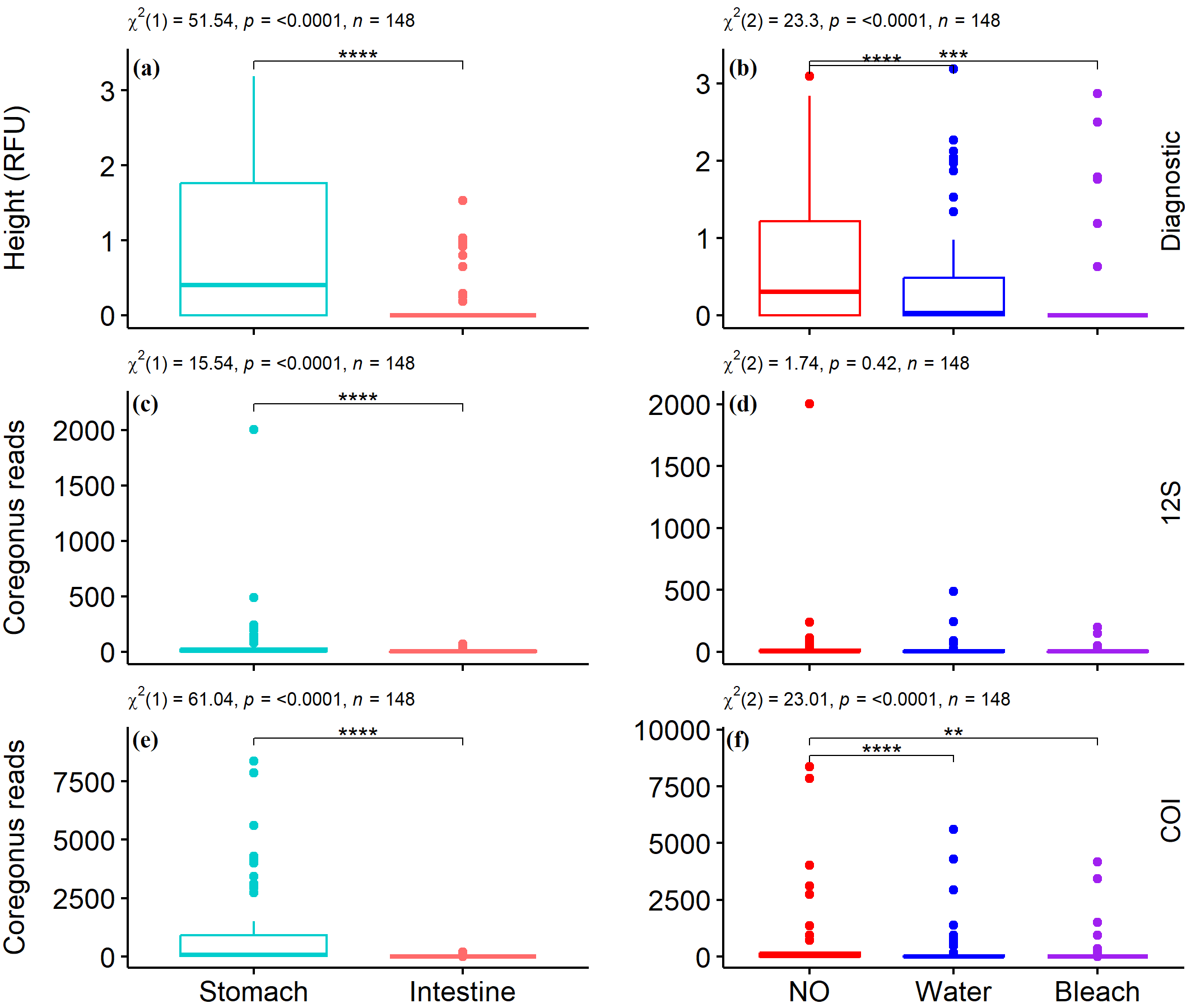


**Figure A5** Summary of Kruskal-Wallis test followed by Dunn’s test for pairwise comparisons. The overall amplification of whitefish DNA between gut types (a) and among cleaning treatments (b) based on diagnostic analysis, and number of reads assigned to whitefish between gut types (c) and among cleaning treatments (d) based on 12S metabarcoding, and whitefish reads between gut types (e) and among cleaning treatments (f) based on COI metabarcoding.


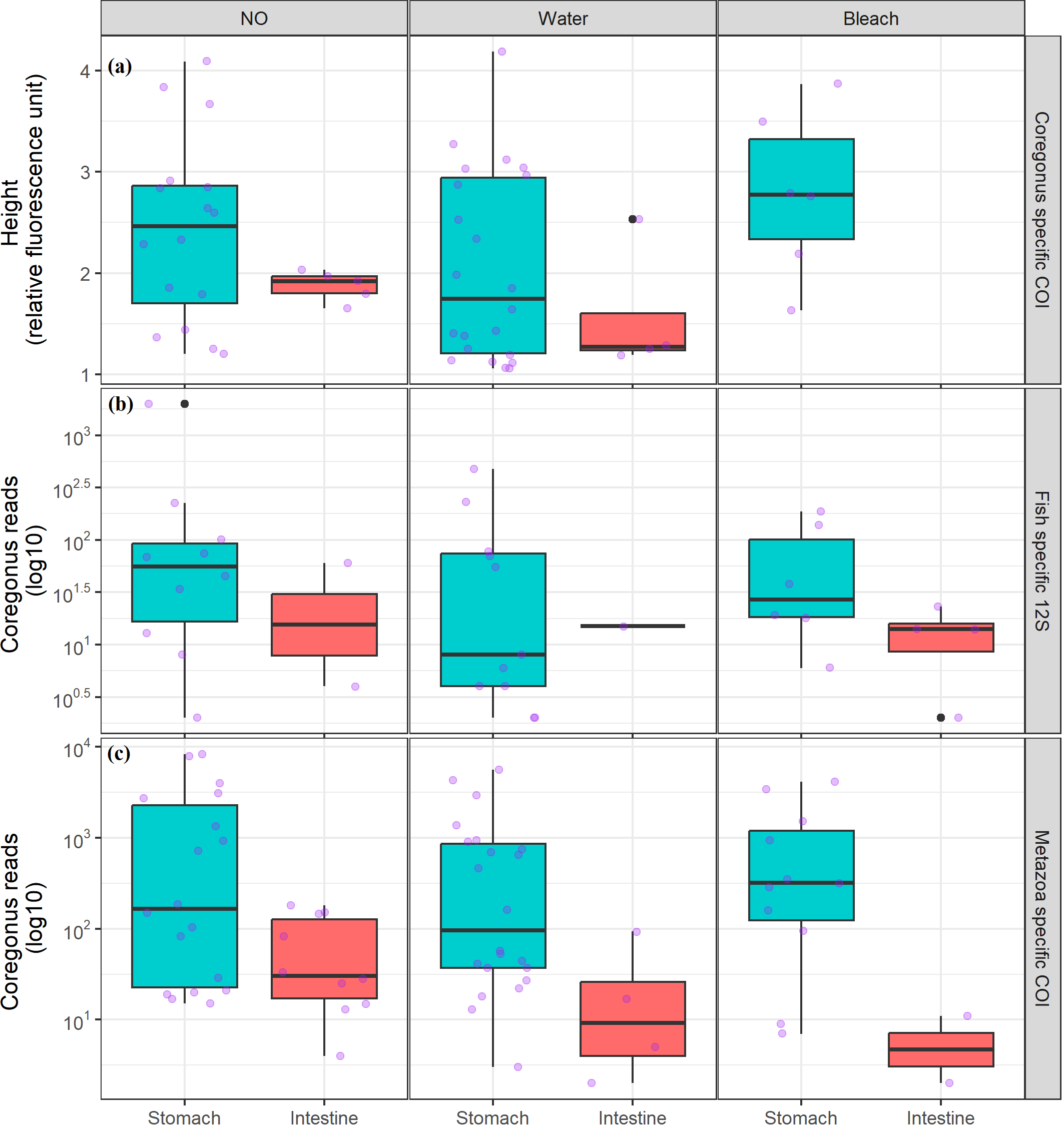


**Figure A6** Amplification of whitefish DNA using species specific (a), 12S (b), and COI (c) primers from the stomach and intestine of redfish with different cleaning treatments excluding zeros. The whitefish DNA reads are based on metabarcoding data.

**
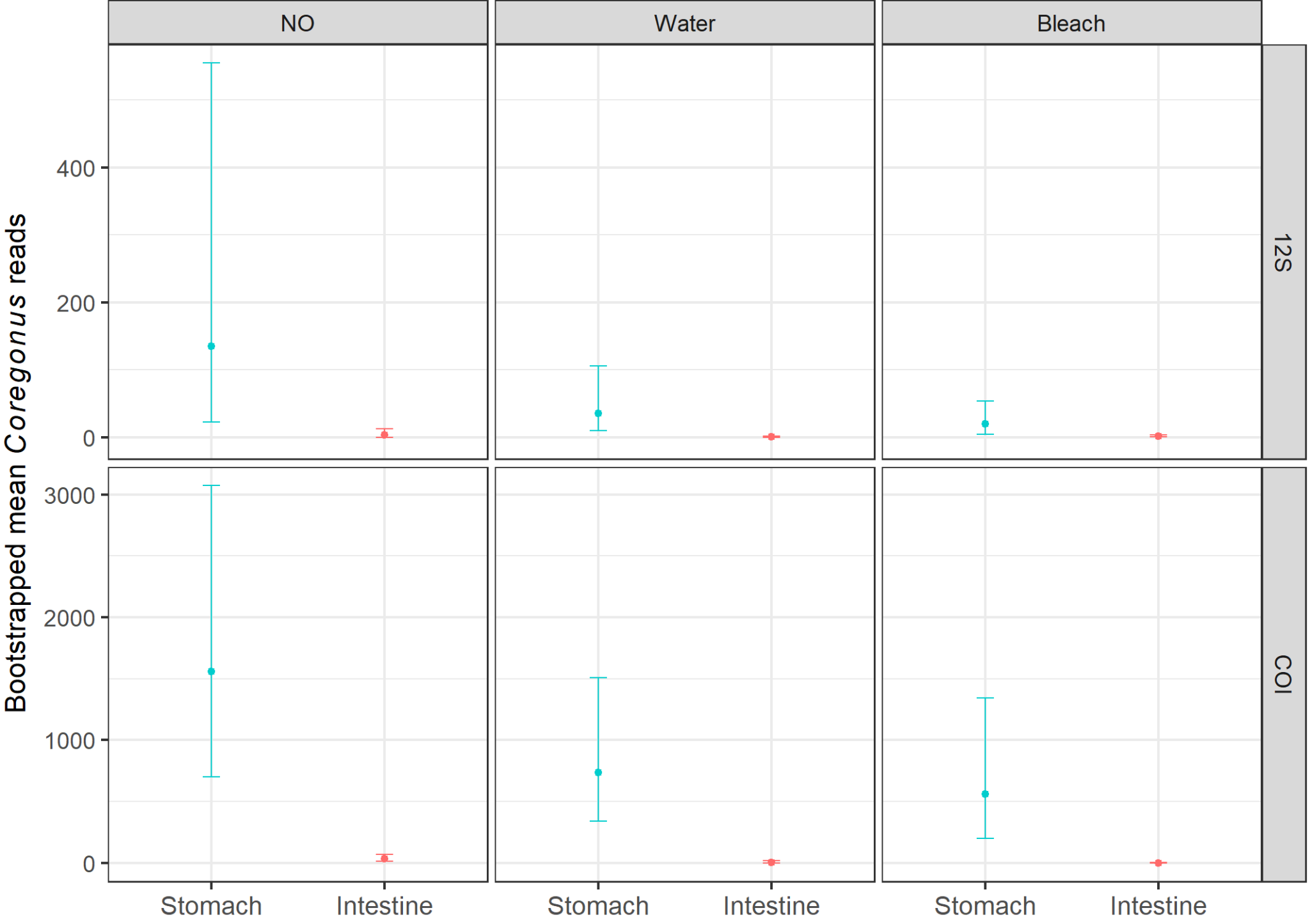
**

**Figure A7** Untransformed 95% bootstrapped confidence interval of mean number of whitefish reads detected by metabarcoding of redfish gut samples with different cleaning treatments using (a) 12S and (b) COI markers.

**Protocol A1:** **Subsampling protocol**

The subsampling protocol is based on the experiences gained during sampling and subsampling of the beaked redfish (*Sebastes mentella*), results of this paper, and ongoing subsampling of Atlantic mackerel (*Scomber scombrus*). We recommend the following steps while acquiring samples for diet analysis, particularly if the aim is to infer the diet components based on molecular approaches.

1. ***Establish subsampling controls in the dissection room and also take swab samples of individual fish surfaces.***

The aim of subsampling and swab controls [(also see O’Rorke et al. 2013)](https://paperpile.com/c/Lri6Dw/nbioJ/?prefix=also%20see) are to keep track of external DNA from the dissection room and body surface of the samples respectively. If there are some common taxa between gut samples, subsampling, and swab controls, the total number of reads assigned to taxa of exogenous origin may gradually decrease with a cleaning gradient making it possible to detect potential contaminants.

1. ***Rinse each sample with sterile water, and 1% sodium hypochlorite (leave it for 5-10 minutes to make it effective), and finally rinse thoroughly with sterile water.***

The first wash by water removes some of the easily removable contaminants from the body surface and further cleaning by bleach degrades and removes additional contaminants from the body surface. Note that bleach may take a while to effectively work. The final rinse with water removes both fragmented DNA and bleach. Note that traces of bleach may degrade DNA if they enter the subsamples.

1. ***Freeze the samples if dissection is not possible in the field or dissect.***

If it is not possible to harvest stomachs and intestines in the field, we recommend freezing fish samples in the field to avoid further degradation of DNA and dissecting the fish in the dedicated dissecting room after they are brought into the lab. It is also recommended to use physically separated facilities for fish dissection and DNA extraction to avoid additional contamination as free-floating environmental DNA may be more frequent in the vicinity of DNA extraction labs.

The decontamination and subsampling can be performed in batches by designing a platform of appropriate size (see image below) that minimises the overall processing time. Once the frozen fishes are separable from the collection batch, they can be laid down in the platform and the decontamination step can be followed for several fishes at once (see image below). Based on our experience, it is easier to dissect and sample gut contents when fishes are semi frozen as the stomach and intestine are still intact. In the defrosted fishes, intestines are disintegrated in high frequencies demanding additional sterile spoons for sample collection. It is crucial to change gloves and use separate sterile dissecting tools for each fish sample.

1. ***After dissection, collect both the stomach and intestine contents in a sterile smasher bag, add appropriate volume of 70-90% ethanol or ATL buffer to make homogenate and homogenise samples by mechanical smasher or by manually massaging the bag.***

Note that mixing stomach and intestine has two advantages: i) it dilutes the quantity of contaminants DNA as we have seen that stomach is more susceptible to contamination than intestine and by mixing both concentration of contaminants will be reduced to nearly half, and ii) it maximises the actual prey capture in case there are some unique taxa in stomach or intestine.

It is important to also note that sample preservation in ethanol demands an additional step to remove ethanol prior to DNA extraction as presence of alcohol may hinder DNA extraction. However, for example, adding ATL reduces two steps compared to DNA extraction from ethanol-preserved samples as we can directly move to the tissue digestion step of the DNeasy Blood and Tissue extraction protocol. Thus, we suggest using ATL for subsample preservation prior to DNA extraction.

1. ***Take subsamples from the homogenate in an appropriate volume and numbers for DNA extraction, and freeze.***

We suggest taking at least two subsamples from each sample for the sake of sample security. Subsample vials completely filled with gut content may burst during freezing, be susceptible to cross-contamination while opening the lids due to spilling, and vortexing may not be efficient to homogenise gut contents while taking subsamples for DNA extraction. Thus, as a good practice we recommend filling ⅔ of the subsampling vials.


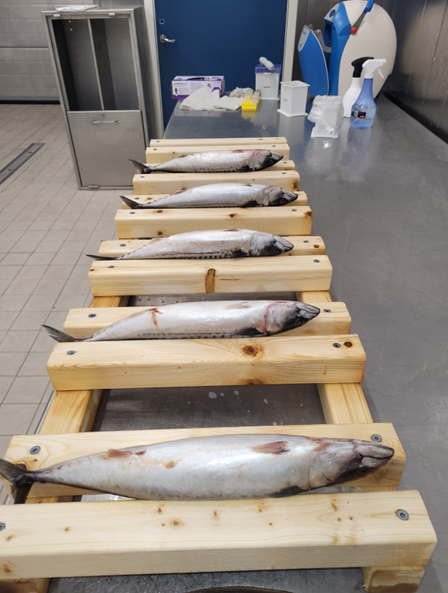


A wooden platform designed to decontaminate and subsample fish samples for molecular diet analysis in batches of six.
